# Supplementary material for: A speed limit on serial strain replacement from original antigenic sin
Source: Proc Natl Acad Sci U S A. 2024 Jun 10;121(25):e2400202121. doi: 10.1073/pnas.2400202121 (PMC11194583; doi:10.1073/pnas.2400202121)
Supplement: Supplementary file 1 — Appendix 01 (PDF) [file pnas.2400202121.sapp.pdf]

Supporting Information for

A speed limit on serial strain replacement from original antigenic sin

Lauren McGough and Sarah Cobey

Lauren McGough
E-mail: mcgough@uchicago.edu

This PDF file includes:

Supporting text
Figs. S1 to S2
SI References

Supporting Information Text

More details on serial strain replacement assumptions. We expand on the assumptions underlying the serial strain replacement model, which are nontrivial for a model of OAS and trade full information on the individual scale for tractability on the population scale.

Given complete information about the memory profile of every individual, an individual's protection against a strain $\vec{s}$ could be calculated, but with only the density $h(\vec{s}, t)$ available, we must approximate the distribution of protection. We make a homogeneity assumption, i.e., that the average protection against infection is well approximated by the average protection in a system where the $h(\vec{s}, t)$ memories are distributed randomly among individuals, with each individual having memory to a fixed number of strains, m.

The homogeneity assumption precludes direct consideration of dynamics where the population consists of groups of individuals with highly correlated infection histories that differ substantially between groups, such as birth cohorts; however, this kind of structure could be accommodated by introducing a separate memory density for each cohort and specifying transmission dynamics between cohorts.

Similarly, we assume that the average susceptibility is well approximated by the susceptibility when each individual's memory profile is an independent draw of m memories from $h(\vec{x}, t)$, meaning that homogeneity extends to the number of exposures to the pathogen over time (and therefore over antigenic distance).

When the infection distribution $n(\vec{x}, t)$ takes the localized form in Eq. 10 of the main text and the memory and infection distributions satisfy the coupled differential equations Eqs. 8 and 9 of the main text, it is possible to write down an explicit equation for the form of $h(\vec{x}, t)$. In a zoomed-out limit where $x_1 \gg \sigma$ and $x_i \gg b$ for $i = 2, \ldots, d$,

$$h(\vec{x}, t) \approx \frac{mN_h}{v\tau} e^{-(vt-x_1)/v\tau} \Theta(vt - x_1)\delta(x_2)\cdots\delta(x_d), \qquad [1]$$

where N_h is the population size, $\tau = mN_h/N_I$, and Θ is a step function, $\Theta(y) = 1$ for $y \geq 0$ and $\Theta(y) = 0$ for $y < 0$. Eq. 1 follows from main text Eqs. 8 and 9, assuming $n(\vec{x}, t)$ takes the form in main text Eq. 10 (1).

Derivation of blunting neighborhood conditions. We derive the existence of blunting neighborhoods (main text, Fig. 3) from the conditions for the creation of memory in the every-epitope and any-epitope models (main text, Eqs. 3 and 4).

First, the every-epitope model: Let $\vec{p} = (p_1, \ldots, p_d)$ be a reference point in antigenic space. Suppose the strain $\vec{s}$ is in $\mathcal{S}$, and there exists at least one j for which $|s_j - p_j| < \frac{b}{2}$. Suppose the host is infected by (or vaccinated with) strain $\vec{s}'$ that is also within a distance $\frac{b}{2}$ from $\vec{p}$ in dimension j. If both $d_j \equiv (s_j - p_j)$ and $d'_j \equiv (s'_j - p_j)$ have the same sign, then $|s_j - s'_j| = |d_j - d'_j| \leq |d_j| < \frac{b}{2} < b$, and therefore $\vec{s}'$ is not added to $\mathcal{S}$. If $(s_j - p_j)$ and $(s'_j - p_j)$ have different signs, then $|s_j - p_j| + |s'_j - p_j| = |s'_j - s_j| < \frac{b}{2} + \frac{b}{2} = b$, and so $\vec{s}'$ is not added to $\mathcal{S}$. Either way, $\vec{s}'$ will not be added to $\mathcal{S}$, meaning that $\vec{s}$ will remain the only strain in $\mathcal{S}$ within a distance $\frac{b}{2}$ of $\vec{p}$ in dimension j in the every-epitope model.

Then, the any-epitope model: Let $\vec{p} = (p_1, \ldots, p_d)$ be a reference point in antigenic space. Suppose the strain $\vec{s}$ is in $\mathcal{S}$, and for all j, we have $|s_j - p_j| < \frac{b}{2}$. Suppose the host is infected by (or vaccinated with) strain $\vec{s}'$ that is also within a distance $\frac{b}{2}$ from $\vec{p}$ in all dimensions $j = 1, \ldots, d$. Applying the same argument as for the every epitope model to each dimension separately, it follows that $|s_j - s'_j| < b$ for all j, and thus $\vec{s}'$ is not added to $\mathcal{S}$. Thus $\vec{s}$ remains the only strain in $\mathcal{S}$ that is within a distance of $\frac{b}{2}$ of $\vec{p}$ in every dimension in the any-epitope model.

References

1. J Marchi, M Lässig, AM Walczak, T Mora, Antigenic waves of virus-immune coevolution. *Proc. Natl. Acad. Sci. U. S. A.* **118** (2021).

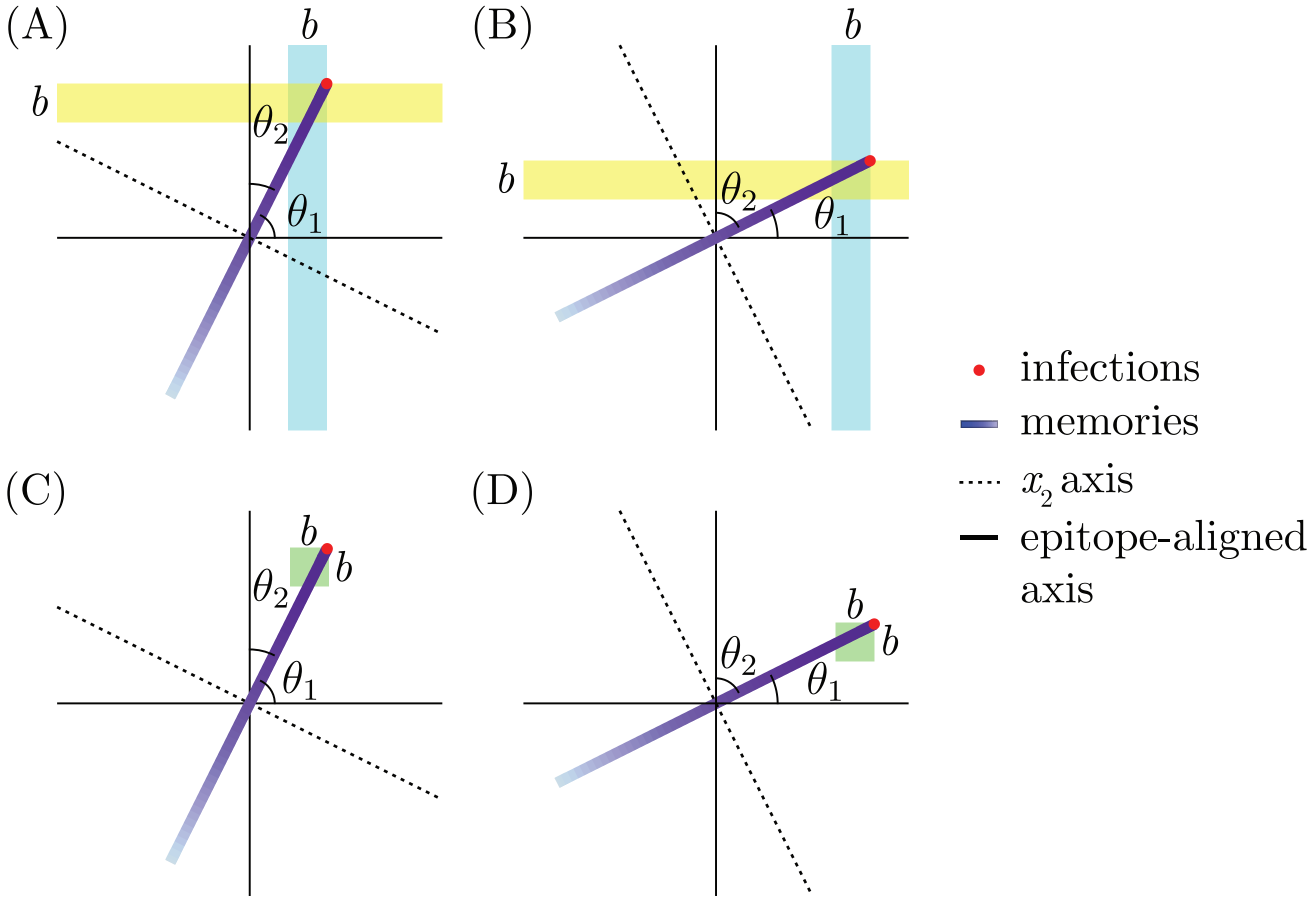

Fig. S1. The slowest (fastest) epitope determines the integration bounds in the every- (any-) epitope model. (A, B) The every-epitope model. (A) The blue stripe is the blunting neighborhood that maximizes the number of memories being integrated over. The interval of x_1 that lies in the blue region has length $b/\cos\theta_1$. (B) The yellow stripe is the blunting neighborhood that maximizes the number of memories being integrated over. The interval of x_1 that lies in the yellow region has length $b/\cos\theta_2$. (C, D) The any-epitope model. The green regions are the blunting neighborhoods that maximize the number of memories being integrated over. The length of the x_1 interval that lies in the green region equals $b/\cos\theta_2$ in (C), and $b/\cos\theta_1$ in (D).

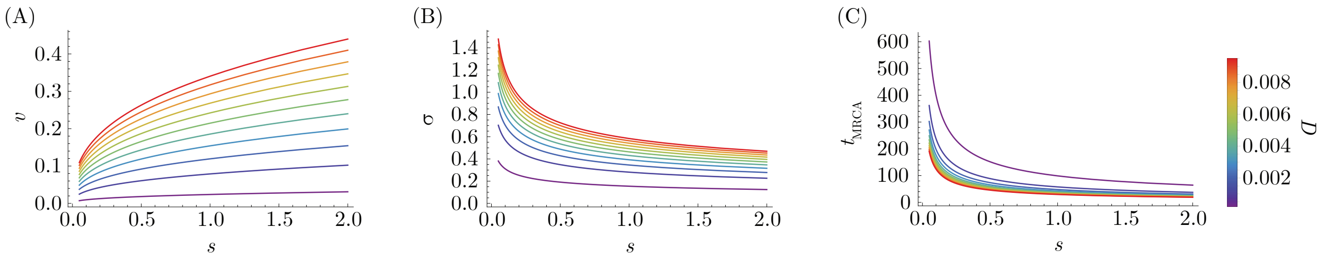

Fig. S2. Dynamical quantities are monotonic as a function of the fitness gradient s. (A) The wave velocity is monotonically increasing, and (B ,C) the infection standard deviation σ as well as the time to most recent common ancestor t_{MRCA} are monotonically decreasing.
